# Alterations in Olfactory Cortex Volume in Mild Cognitive Impairment and Mild Alzheimer’s Disease Dementia: A Study of Sex-Related Differences

**DOI:** 10.3390/brainsci15060610

**Published:** 2025-06-04

**Authors:** Majed M. Alotaibi, Matteo De Marco, Rona Graham, Annalena Venneri

**Affiliations:** 1 Medical Genomics Research Department, King Abdullah International Medical Research Center, King Saud bin Abdulaziz University for Health Sciences, Riyadh 11481, Saudi Arabia; 2 College of Health, Medicine and Life Sciences, Brunel University of London, Uxbridge UB8 3PH, UK; matteo.demarco@brunel.ac.uk; 3 Research Center on Aging, CIUSSS de L’Estrie–CHUS, Sherbrooke, QC 1G 1B1, Canada; rona.graham@usherbrooke.ca; 4 Department of Pharmacology and Physiology, Faculty of Medicine and Health Sciences, University of Sherbrooke, Sherbrooke, QC J1N 3C6, Canada; 5 Department of Medicine and Surgery, University of Parma, 43121 Parma, Italy

**Keywords:** Alzheimer’s disease, neuroimaging, olfactory cortex, MRI

## Abstract

Background/Objectives: Aging is one of the greatest risk factors for neurodegenerative diseases such as Alzheimer’s disease (AD). As the disease progresses, neural loss in brain regions, such as the olfactory cortex (OC), i.e., a set of areas including the mediotemporal and orbitofrontal regions, may lead to dysfunction in the sense of smell and affect other brain regions that relate to the olfactory cortex by either afferent or efferent projections. Methods: The objective of this study was to assess sex-related differences in olfactory cortex volume using magnetic resonance imaging in individuals with mild cognitive impairment, probable dementia of the AD type and in healthy older adults, using the Mini-Mental Statement Examination score, years of education, and total intracranial volume as correction factors. Results: Atrophy of the olfactory cortex was observed in patients of both sexes with probable AD dementia. However, at the MCI stage, significant volumetric loss in the OC was detected in females only but not in males. Conclusions: This finding indicates greater pathological effects in this region in females at an earlier disease stage than in males. This study suggests that OC volume loss occurs differently between the sexes in older adults, with volumetric loss being greater in females.

## 1. Introduction

Alzheimer’s disease (AD) is a neurodegenerative disorder of the central nervous system that affects the aging population. Indeed, it is one of the most common progressive neurodegenerative disorders causing gradual cognitive decline in the older adult population and one of the most common medical conditions in the world [1,2]. The prevalence of this age-related neurodegenerative disease is increasing dramatically concurrently with life expectancy. This disease causes cognitive deterioration, sensory dysfunction (e.g., olfactory dysfunction), and brain regional atrophy [3], among other symptoms and difficulties.

This prevalent and challenging condition leads to dementia by impairing a person’s cognitive and sensory abilities that are essential for daily tasks. The prevalence of this disease affects older adults across all societies globally, with its effects having a range of societal impacts on caregivers and healthcare systems worldwide. AD is a multifactorial disease associated with several risk factors [4,5]. The main hallmarks of AD include extracellular senile plaques of β-amyloid and intracellular neurofibrillary tangles of misfolded tau protein [6,7]. AD is characterized by a range of risk factors, including aging, the presence of the Apolipoprotein E allele ε4 (APOE ε4), family history, low physical activity, and a low level of education [8,9,10,11]. Importantly, not all individuals with one or more of these risk factors develop this disease. In addition to the deposition of β-amyloid and tau protein, the pathology of AD also manifests with inflammation, caspase activation, neurogenesis decline, and neurotransmitter deficits [12,13,14,15].

In mild cognitive impairment (MCI), several aspects of memory are impaired, including episodic, working, and semantic memory [16]. The pathological processes of AD also lead to brain atrophy, with significant reductions in the medial temporal regions and subsequent volumetric losses in the cortical temporal and parietal regions [17]. Volumetric reduction of olfactory-related brain regions is also detectable and quantifiable at the MCI stage-considered the “pre-dementia” stage of AD [18]-and it is more severe at the dementia stage [19].

There is reported evidence that olfactory dysfunction is observed in AD and that is an early symptom of the disease [20,21]. Moreover, different publications have shown atrophy of the olfactory brain regions [22,23,24], particularly in the olfactory cortex (OC) [25,26]. Evidence of a reduction in the volume of olfactory-related regions at the MCI stage has also been reported [19]. It is important, however, to differentiate between physiological age-related decline and that consequent to a neurodegenerative form of MCI [8], as it has been found that 68% of MCI cases progress to AD at approximately 28 months of follow-up [27].

Magnetic resonance imaging (MRI) has been extensively used to assess brain changes at various stages along the AD continuum. In detail, MRI, especially T1-weighted (T1W) structural scans, can detect regional atrophy, and at high field, it is possible to obtain a quantitative measure of global and regional volume loss even in specific hippocampal subfields [28]. Significant volume loss in these hippocampal regions is detectable quite early at the MCI stage and more globally throughout the cortex, and it is even more noticeable in patients with established AD dementia [29]. Although MRI features have been extensively studied for their potential contribution to the early detection of AD (see [30] for a recent study that investigated the utility of an extensive set of radiomic features for early detection of AD), few studies have focused on the volumetric assessment of the olfactory cortex among patients with MCI and AD and compared olfactory cortex volume (OCV) with that of healthy older adults, despite evidence from behavioral studies suggesting that olfactory dysfunction can be found early in the AD course [21]. In this study, we investigated OCV in MCI and mild probable AD dementia patients and in an age-appropriate control group using structural MRI with a specific focus on sex-related differences. The association between participant demographics and OCV was also investigated. We hypothesized that there would be a reduction in OCV across groups and that an effect of the APOE ε4 allele and education on patients’ OCV would be present. The aim of this study was to ascertain whether OCV loss were the outcome of age-related decline or whether OCV loss were fostered by AD-related neurodegeneration.

## 2. Materials and Methods

### 2.1. Participants

Healthy older adult participants and individuals with a diagnosis of AD were selected from the Sheffield Ageing Database, coordinated by the Department of Neuroscience at the University of Sheffield, United Kingdom [31]. This database includes several hundreds of participants in research projects who have agreed to make their assessments available for additional research studies related to the optimization of early detection and characterization of AD. The participants included in this database were recruited between June 2011 and July 2016 and were aged between 55 and 88 years, with an overall mean age of 73.9 years and a standard deviation of 5.8 years. A total of 213 participants were retrospectively selected based on their clinical profile and availability of relevant assessments: 95 cognitively healthy older adults (age range: 66–85), 81 individuals with MCI (age range: 58–86), and 37 participants with mild probable AD dementia (age range: 55–88). Participants were selected among those who had consented to their data being retained in an anonymized database and used for additional research, had a three-dimensional T1W MRI scan available, had a comprehensive neuropsychological assessment, and had at least 4 years of clinical follow-ups to support initial clinical diagnosis. All available participants fulfilling these requirements were included. A power analysis determined that our subsamples were of adequate size to achieve a power of 0.08 with an alpha level of 0.05 and a moderate effect size of 0.5 with a between group allocation ratio of 0.45. The assessment battery included the Mini-Mental State Examination (MMSE) and tests of short-term and working memory, episodic memory, lexical–semantic processing, executive functions, and visuoconstructive abilities (see [32,33] for details). Eligible participants in the healthy older adult control group were those with no neurological conditions or cognitive decline based on their scores on different cognitive tests within the normal age range, and they represented an opportunity sample of individuals recruited as healthy participants in several research projects carried out by two of the authors (AV and MDM). Participants with MCI were diagnosed using Petersen’s diagnostic criteria and Albert et al.’s criteria [8,34], while mild probable AD dementia patients were diagnosed using McKhann et al.’s clinical diagnostic criteria [35]. All participants completed a 1.5 T MRI protocol, including a T1W MRI brain scan. For some participants in the patient subgroups, their APOE ε4 status was available. There were 59 patients for whom APOE status was known, and these included 22 APOE ε4 carriers (1 APOE ε4/ε4 and 21 APOE ε4/ε3) and 37 APOE ε4 non-carriers. In addition to age, MMSE score and years of education were also noted, given their relationship with brain regional volume [36,37,38].

All participants provided written informed consent at the time of initial recruitment. Ethical approval for this retrospective data analysis was obtained from the West Scotland Regional Ethics Committee 5 (Ref No: 19/WS/0177, 21 November 2019). All procedures based on the participants’ clinical assessments for this study were in line with the Declaration of Helsinki (1964) and followed institutional ethical standards.

### 2.2. MRI Acquisition and Processing

**Figure 1:**
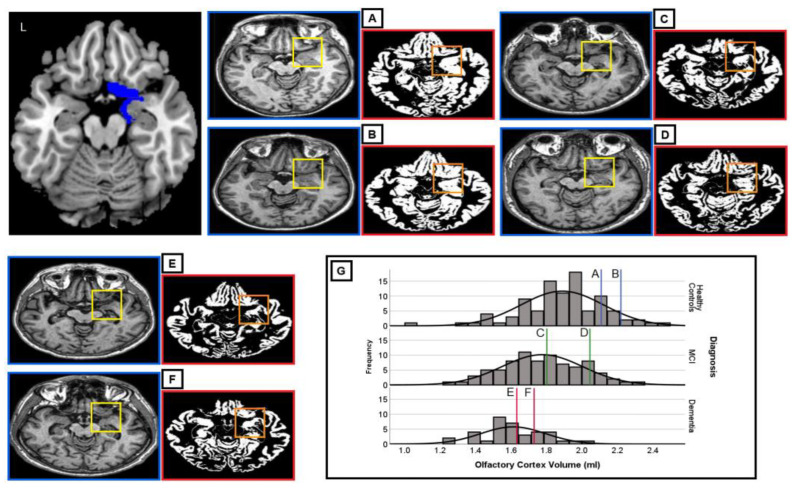
Graphical illustration of the olfactory cortex map used in individual volumetric extractions (L = left). The olfactory cortex is illustrated in the top left (in blue) and is visually identifiable in the framed portion of each axial slice, i.e., (**A**–**F**) native-space images (yellow frame) of the original T1W MRI scans and of the segmented gray matter maps (orange frame) of the olfactory cortex in a case series extracted from the study cohort are shown. The olfactory cortex is highlighted only in the right hemisphere in these images. (**G**) shows the distribution of OCV scores in the three subgroups; vertical lines indicate where each of the six participants is located within its diagnostic subgroup. The diagnostic and demographic characteristics of these six study participants are as follows: (**A**) female, 66 years, 7 years of education, Mini-Mental State Examination (MMSE) = 29, diagnosis: healthy older adult control, olfactory cortex volume (OCV) = 2.221 mL; (**B**) male, 66 years, 8 years of education, MMSE = 27, diagnosis: healthy older adult control, OCV = 2.109 mL; (**C**) female, 81 years, 10 years of education, MMSE = 25, diagnosis: mild cognitive impairment, OCV = 1.803 mL; (**D**) male, 64 years, 11 years of education, MMSE = 30, diagnosis: mild cognitive impairment, OCV = 2.046 mL; (**E**) female, 69 years, 5 years of education, MMSE = 22, diagnosis: dementia, OCV = 1.738 mL; and (**F**) male, 75 years, 5 years of education, MMSE = 23, diagnosis: dementia, OCV = 1.633 mL.

Three-dimensional T1-weighted brain scans, acquired as part of an extensive MRI protocol, were used for each participant. These were Turbo Field Echo T T sequences acquired on a Philips Achieva 1.5 T scanner equipped with a SENSE head coil. Statistical Parametric Mapping (SPM) software, version 12 (Wellcome Centre for Human Neuroimaging, London, UK) was used for processing all MRI images in a Matlab R2016b environment, version 9.1 (Mathworks Inc., Natick, MA, USA). Following the most up-to-date voxel-based morphometry pre-processing pipeline, all images were initially segmented to separate three tissue maps: gray matter, white matter, and cerebrospinal fluid. The pre-processing and volume extraction procedures have been detailed in a previously published article by our team [31]. In brief, all gray matter maps were also modulated, normalized to the T1W template available in SPM, and smoothed with an 8 mm full-width at half-maximum isotropic Gaussian kernel. Global volumes for all tissue classes obtained from each scan in the native space were quantified using the “get_totals” script (http://www0.cs.ucl.ac.uk/staff/g.ridgway/vbm/get_totals.m, access date 5 November 2024). Total intracranial volume (TIV) was calculated as the combined global volume of gray matter, white matter, and cerebrospinal fluid. The region of interest (ROI) was defined using the SPM12 toolbox Wake Forest University PickAtlas [39] that was used to specify olfactory-related brain regions, as detailed in the Automated Anatomical Labeling atlas of the human brain, used to create an olfactory cortex mask in the Montreal Neurological Institute (MNI) space [40]. This automatic extraction procedure was used to obtain the volumetric information of this ROI in each brain hemisphere to have a total OCV value. Figure 1 provides a graphical illustration of the map of the olfactory cortex ROI used to extract individual OCV. Sample images of individual axial slices and relative gray matter maps are provided for representative individuals in each subgroup and sex.

### 2.3. Data Analysis

Statistical analyses were carried out using the IBM SPSS software version 26 (IBM SPSS Statistics for Windows, IPM Corp, Armonk, NY, USA) and GraphPad software version 9 (GraphPad Software San Diego, CA, USA). The characteristics of the study cohort were defined using descriptive statistics. We assessed whether the OCV and TIV were normally distributed. To explore the data and answer the study questions, one-way ANOVAs were run to compare regional brain volumes among the three groups. Moreover, an analysis of covariance (ANCOVA) was used in the form of a Univariate General Linear Model to regress out the influence of TIV and educational level. Pearson’s correlation coefficient was used to test for any possible association between OCV and any of the covariates. To determine the influence of the APOE ε4 risk factor, a *t*-test was carried out to compare regional volumes of carriers and non-carriers for those patients for whom APOE status was known to evaluate the effect of genotype on OCV. The statistical threshold of significance for OCV comparisons was set at *p* < 0.001.

## 3. Results

### 3.1. Demographics of the Participants

No significant differences in age were found across the three groups (both sexes, *p* = 0.33, females *p* = 0.09; males *p* = 0.36). There were significant differences in education (both sexes: *p* = 0.001; females: *p* = 0.02; males: *p* = 0.01) and MMSE (both sexes: *p* = 0.0001; females: *p* < 0.0001; males: *p* < 0.0001) (Table 1). *Post-hoc* comparisons were carried out to characterize these effects in more detail. Both females and males in the control group had significantly higher education levels than female and male participants with probable AD dementia (*p* = 0.02 and *p* = 0.01, respectively). As for MMSE scores, significant differences were found for both sexes, between controls and MCI individuals, between controls and participants with probable AD dementia, and between MCI individuals and participants with probable AD dementia, that is, females: *p* = 0.01, *p* < 0.0001, and *p* < 0.0001, respectively; males: *p* = 0.01, *p* < 0.0001, and *p* < 0.0001, respectively.

### 3.2. Olfactory Cortex Volume

**Figure 2:**
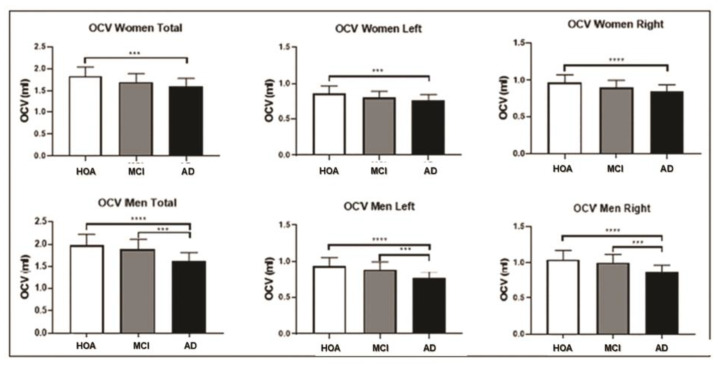
Olfactory cortex volume (OCV) differences among the three groups: comparisons among the three groups are shown for the female population (above) and male population (below). Error bars indicate standard deviations. The significance level was set at *p* < 0.001. (*** *p* ≤ 0.001 and **** *p* ≤ 0.0001). HOA = healthy older adults; MCI = mild cognitive impairment; AD = probable AD dementia.

Considering the overall participants together, the OCV was normally distributed. One-way ANOVA showed significant differences in OCV volume among the three groups (F = 21.18, DF = 2.210, *p* < 0.0001) (Figure 1). Bonferroni-corrected *post-hoc* comparison tests showed that there were significant differences in the volume of this structure for both females and males when the probable AD dementia group was compared with the healthy older adult controls and for males only when the AD dementia group was compared with the MCI group (Figure 2, Table 2).

Differences remained significant after covarying for age (females F = 9.979, DF = (2, 119), *p* = 0.0001, males F = 14.445, DF = (2, 86), *p* = 0.0001), education (females F = 9.853, DF = (2,119), *p* = 0.0001, males F = 10.451, DF = (2, 86), *p* = 0.0001), or TIV (females F = 9.039, DF = (2, 119), *p* = 0.0001, males F = 20.786, DF = (2, 86), *p* = 0.0001). When adjusting for MMSE, the differences in OCV among the three groups were no longer significant in the female and male subgroups (females F = 4.223, DF = (2, 119), *p* = 0.017, males F = 1.918, DF = (2, 86), *p* = 0.153), and all pairwise testing showed no significant differences in the comparisons of healthy controls with MCI, healthy controls with probable AD dementia, and MCI with probable AD dementia. MMSE accounted for differences in OCV when comparing the three groups: healthy controls, MCI, and probable AD dementia.

Males with probable AD dementia showed smaller volumetric values for the total, left, and right OCV than males with MCI. Pearson’s correlation showed no significant association between OCV and age, except for males in both the MCI and probable AD dementia groups, with significant negative associations found in both groups (*p* = 0.04 for both groups). Education was positively associated with OCV only in males in the MCI group (*p* = 0.01). A positive association was found between MMSE scores and OCV in the female control group only (*p* = 0.01).

To determine whether differences between males and females occurred at different disease stages, the OVC of healthy older adult controls was taken as a reference, and deviations from this group were calculated for males and females at different disease stages. In the MCI group, females showed a smaller OCV than males, with reference to the volume of the healthy older adult controls. The difference in percentage of OCV was higher in females (left 11%, right 9%, and total 12%) than in males (left 3.2%, right 3.8%, and total 5%), while in probable AD dementia cases, the difference in percentage of OCV was lower in females (left 16.6%, right 15%, and total 11.5%) than in males (left 19.4%, right 17.3%, and total 19%).

### 3.3. APOE ε4 Carriers vs. APOE ε4 Non-Carriers

T-tests comparing APOE ε4 allele carrier and non-carrier patient subgroups showed no significant differences in the total, left, and right OCV in either female or male participants (Table 3).

## 4. Discussion

This study investigated whether sex-related volumetric differences in the olfactory cortex volume were detectable between healthy older adult controls and patients at different stages of cognitive decline of probable AD etiology (i.e., MCI and mild probable AD dementia). In addition, the effect of carrying the APOE ε4 allele on volumetric measures of the olfactory cortex was investigated in a subgroup of participants for whom APOE ε4 status was known.

The findings in this cohort showed that there were differences in the volume loss in the olfactory cortex between females and males. In the subgroup of females, there was a significantly greater loss in the total, left, and right OCV in participants with probable AD dementia when compared with healthy older adult controls, while no significant volume loss was found in the comparison between the mild probable AD dementia and MCI subgroups. This indicates that significant OCV loss is clearly detectable in females at the MCI stage, and any further volume loss from MCI to mild probable AD dementia is negligible. In contrast, in the male subgroups, there was a significant difference between the OCV of the MCI patients and that of the mild probable AD dementia patients, indicating that volumetric loss in this region in males may be more gradual and distributed across the stages of disease severity. In support of this, the difference in OCV between males at the MCI stage and healthy older adult controls was minimal, whereas it became more accentuated at the mild probable AD dementia stage. Overall, these findings indicate a greater and earlier impact of AD pathology on OCV in females than in males.

It is worth mentioning that the effect of physiological aging processes on OCV in healthy older adult controls has already been established, and there is evidence from previous findings that OCV in older adults undergoes significant shrinkage when compared with middle-aged and young individuals [31].

The olfactory cortex is defined as a region that receives projections from the olfactory bulb. The olfactory bulb is one of the structures that are first affected in AD [21], and deterioration of olfactory regions has been reported to reflect early regional accumulation of neurofibrillary tangles [41]. Moreover, there is evidence that the accumulation of β-amyloid plaques and neurofibrillary tangles occurs in the extended olfactory system and its connected regions [42] and that in AD, there is elevation of tau deposition in the piriform cortex [43].

When inspecting the magnitude of loss in the left and right hemispheres, the left OCV showed greater differences than the right. This finding supports previous evidence in the literature that the left hemisphere of the brain is affected by greater volume loss earlier in the course of AD, with a greater effect of AD pathology on the left side early in the disease course than in the right hemisphere [44,45,46]. Greater atrophy in the left hemisphere has been interpreted as reflecting the early stage of AD pathology in the brain [47] with subsequent spreading across the cortex and hemispheres, with observations of greater left-sided accumulation of β-amyloid and/or tau [48].

In the present study, the influence of the APOE ε4 allele was assessed in a subgroup of patients with known APOE status. The findings show that carrying the APOE ε4 allele did not influence OCV, as no significant differences in OCV were observed between APOE ε4 carriers and APOE ε4 non-carriers. This finding is not in line with previous evidence from studies that reported that APOE ε4 carriers show greater brain atrophy than non-carriers [49,50]. However, this discrepancy in findings should be interpreted with caution because the number of participants in the current study for whom a genetic profile for the APOE gene was available was very small. Furthermore, patients at different stages of the disease were analyzed together, as the small numbers did not allow stratification by APOE status and disease stage. Heterogeneity in disease stage could have masked any possible effect due to this risk gene.

No strong body of research has investigated the impact of aging, MCI, and mild probable AD dementia on OCV. The majority of research has been carried out to investigate other olfaction-related brain regions such as the olfactory bulb. The findings of the present study highlight the critical need to investigate the full range of olfactory brain regions to understand in more detail how they are progressively vulnerable to AD pathology.

This study has some limitations. The cross-sectional design is a limitation, as it would be more appropriate to investigate atrophy of the olfactory regions longitudinally to quantify volume loss over time in line with progression through the different stages of the disease. However, having included healthy older adult controls, an MCI group, and a mild probable AD dementia group, therefore covering the spectrum of this disease, might have minimized the limitations of a cross-sectional approach.

Additionally, the effect of APOE could not be fully clarified given that not all people in the sample had agreed to genetic testing, adding, therefore, an additional limitation to this study.

One final limitation is that our sample was diagnosed using clinical criteria with no evidence of AD pathology based on fluid biomarkers or molecular imaging. This sample was recruited prior to the suggestion that biological evidence of AD pathology is needed for an in vivo diagnosis [51]. However, the availability of extensive assessments, a pattern of neurodegeneration typical of AD, and a long history of clinical follow-ups mitigate the risk of potential diagnostic errors.

## 5. Conclusions

In conclusion, the findings of the present study indicate that olfactory-related brain regions show volumetric decline in AD and MCI, and this reduction is more accentuated and appears at an early disease stage in females. These findings suggest that a test of olfactory function should be incorporated into clinical assessment, and it might also be advisable to measure the volume of olfactory regions in the aging population at risk as a possible indicator of incipient AD. Finally, the findings also indicate that sex is a factor that should be considered in the clinical evaluation of patients, as males and females appear to have differences in the effects of disease on the olfactory cortex, and any interventions should be tailored by taking individual sex differences into account.

## Figures and Tables

**Table 1:** Demographic characteristics of the participants.

	Total Number	Females				Males			
		N		Mean (SD)		N		Mean (SD)	
			Age	Education	MMSE		Age	Education	MMSE
Healthy Older Adults	95	57	72.4 (4.3)	10.3 (3.9)	28.5 (1.7)	38	73.4 (4.9)	12.7 (4.8)	28.4 (1.4)
MCI	81	47	73.9 (6.8)	8.9 (3.2)	27.4 (1.9)	34	74.9 (5.3)	11.5 (4.2)	27 (1.9)
Probable AD Dementia	37	19	75.7 (7.7)	7.6 (3.6)	20.7 (2.1)	18	75.3 (6.8)	8.6 (4.5)	21.2 (2.7)

Note: only a subset in each patient diagnostic category had APOE status available: MCI (16 carriers/26 non-carriers) and probable AD dementia (6 carriers/11 non-carriers). MCI = mild cognitive impairment; AD = Alzheimer’s Disease.

**Table 2:** Significant differences in volume in the olfactory cortex observed in female and male groups.

		HOA vs. MCI Mean Diff. (*p*-Value)	HOA vs. AD Mean Diff. (*p*-Value)	MCI vs. AD Mean Diff. (*p*-Value)
Olfactory Cortex Volume	Females			
	Total F = 11.5 (*p* < 0.0001)	0.13 (0.002)	0.22 (0.0001)	0.08 (0.31)
	Left F = 10.7 (*p* < 0.0001)	0.06 (0.003)	0.11 (0.0002)	0.04 (0.37)
	Right F = 11.7 (*p* < 0.0001)	0.07 (0.002)	0.12 (<0.0001)	0.05 (0.27)
	Males			
	Total F = 15.3 (*p* < 0.0001)	0.09 (0.32)	0.36 (<0.0001)	0.27 (0.0003)
	Left F = 15.9 (*p* < 0.0001)	0.05 (0.25)	0.18 (<0.0001)	0.13 (0.0003)
	Right F = 14.04 (*p* < 0.0001)	0.04 (0.41)	0.18 (<0.0001)	0.14 (0.0005)

(Significance threshold set at *p* < 0.001). HOA = healthy older adults; MCI = mild cognitive impairment; AD = probable AD dementia.

**Table 3:** Comparisons of olfactory cortex volume between APOE ε4 carriers and non-carriers showing no significant differences.

	OCV	
	**t**	***p*-Value**
Females total	0.26	0.79
Females left	0.34	0.73
Females right	0.17	0.86
Males total	1.18	0.24
Males left	1.16	0.25
Males right	1.19	0.24

APOE ε4 carriers (females = 12, males = 10) and non-carriers (females = 19, males = 18).

## Data Availability

Data are available from the corresponding author upon reasonable request.
